# What does a clinically-scientific professional society stand for?

**Published:** 2016-06-27

**Authors:** J Gerris, W Ombelet

**Affiliations:** Genk Institute for Fertility Technology, ZOL Hospitals, Schiepse Bos 6, 3600 Genk, Belgium.; Genk Institute for Fertility Technology, ZOL Hospitals, Genk, Belgium.

**Keywords:** Scientific society, clinical research, congress organization, scientific journal, gynaecological practice

## Abstract

Originated as a mainly social group of befriended colleagues, the VVOG has evolved over the past 55 years to become a truly professional society facing successfully such diverse challenges as organizing scientific congresses, postgraduate training, ethical debates, hands-on training courses, social events, interactions with national and international sister societies but also with the industry, insurers, the government, politicians and patient organisations.

When one considers the mere title of this contribution, one cannot fail to notice four quite different ingredients: the clinic, science, the profession and a society (people).

Does one behold then a mythical animal with a horse’s body, a cow’s head, deer legs and a lions’ tail? Can a scientific society be taken serious if at the same time she defends professional interests? How can research be conducted in an eminently clinical field of work? Is there not some degree of incompatibility, perhaps of antagonism, moving each of the four wheels into another direction, leaving the car spinning around it? The question is not just rhetoric, if you ponder there appears to be a seeming contradiction, a mutual exclusion. The attitude of the scientist, the way he or she stands in the world and in the observable reality is quite different from that of the deed-oriented clinician. The former enjoys unravelling mechanisms and correlations; searching for a wider context he interprets facts as emanations of universal rules and laws. The latter masters the art of dealing with the particular and small but significant differences. He is to the point and acts solution oriented when a person is looking for care and help. He executes what others have listed up and veri ed for him. But both are intelligent persons, making a life, enjoying hobby’s an indulge in all kinds of human activities. Perhaps the former works in a large hospital with an academic vocation and research programs, focused on innovation, research and teaching; the latter works in a private practice or a purely care-oriented hospital and is focused on care in all its human aspect. In reality these two apparent opposites are in some degree present in each one of us and it can only be hoped for that each ends up in a professional context where one fits and thrives.

My personal observation is that the Flemish Society of Obstetrics and Gynaecology (VVOG) is a very mature society giving form and content to these different challenges in a much equilibrated way.

Before anything else the VVOG is a society of and for clinician. I do not think there is even one active VVOG member who does not exert clinical practice. Differences of intensity, focus and degree of idealism may be wide but exposure to patients is an outstanding common characteristic. The much-used expression “the patient stands central” is a reality for all VVOG members.

The VVOG is a scientific society in two meanings of the word: a fundamental and a methodological one. The era during which scientific research was taken seriously only when the investigator was spending solitary days in a dark laboratory room peering long hours through a microscope is long gone. Modern research is conducted in collaborating teams using common space and sharing instruments. It is a full-time occupation not to be combined with a full-time clinical practice. Some of us lead or participate in such a research team. This research answers relatively fundamental questions preceding clinical applications. The other type of science in our professional field pertains to the application of the scientific method to clinical challenges. This is the field of evidence-based medicine and evidence based nursing. The whole philosophy behind the Cochrane collaboration serves this purpose both in case of diagnostic testing and therapeutic interventions. Without conferring absolute truth and value to EBM and EBN, it may be argued that the deep infiltration of both into our daily way of working and thinking may have contributed more to an increase of the quality of our work that all JCI’s, NIAZ and Qmentums combined, or in a different way perhaps.

The primarily clinical-scientific dimension of the VVOG is demonstrated by the many didactic activities we organise in the curse of a year, e.g. the Year Congress, the Spring Meeting, the Ethics & Economy meeting, the postgraduate study days alternating between universities, the courses, the perinatal evenings etc. There are any administrative meetings: 4 physical board meetings, 2 physical scientific board meetings per year, several teleconferences, and board meetings of the working groups and of the special interest groups. The VVOG is allegedly the most active clinical-scientific society in the whole of Belgium.

Not all clinical scientific societies harbour a professional defence commission within their structure, as is the case in the VVOG. Often, the “scientific” and the “professional” are separate entities not being part of one and the same society, but operating quite independently. In the case of separate entities, the professional group can voice more extreme points of view that do not compromise the scientific sister group, which may be an advantage when dealing with political structures. A disadvantage resides in the lack of deep integration and a certain loss of credibility if they operate together. However, when scientific rigour and professional respectability are strived for by one and the same organization, it becomes quickly clear that both have to be in equilibrium with each other and that ethical and economical aspects of our professional are strongly interwoven.

Our maturity as a society is challenged on many ways, but before anything in trying to find a stable equilibrium between science, professional interests and social activities. In addition, the VVOG does not exist in splendid isolation but interacts with many players, e.g. government institutions, insurers, the corporate world, patient organizations and societies of other specialists and midwives, who may have overlapping interests. Evidence based medicine is also sought after in political gremia.

The VVOG maintains renewed contacts and shares activities with the French-speaking gynaecologists from the south of Belgium, the GGOLFB (Groupement de Gynaecologies et Obstétriciens de Langue française de Belgique) and form a functional Royal Belgian Society of Obstetrics and Gynecology, with the recent intention to organize a three-annual national congress.

There is also the international dimension. The VVOG maintains strong contacts, either through individual members or as a society, with national societies of neighbouring countries or with larger international societies such as FIGO, IFFS, ESHRE, ESE, ESCO, ESGO. The VVOG operates a third world cell, stimulating international projects in third world countries. It publishes on a yearly basis the scientific publications by its members and runs two journals. One is the scientific journal called Facts, Views and Visions in ObGyn, the other is a low threshold communication journal called Gunaikeïa. We also have honorary members and there is a special interest group for retired gynaecologists, the VVOG +.

Last but not least the VVOG reserves great attention to two groups that have not been mentioned enough yet. One are the young trainees and the other one are our patients. The trainees do have a representative in the Board, who has no official voting power but who has the floor to ventilate wishes or complaints. This group undergoes a very quick feminization and one of the major challenges for our society is to have more female gynaecologists in board functions. Within the next 10 years, they will form the large majority of the VVOG. For “the” patient it may still be too early to have a structural representation in the board. But constructive interactions with an increasing number of professional patient organisations will quickly increase and we do think about the creation of a new function of “patient ambassador”. The “lead case” are our recent talks with representatives from the Pelvic Floor Problems group, who focus on complications after insertion of vaginal meshes.

This very short and incomplete enumeration of structures and tasks illustrates that the VVOG, under the impulse of energetic chairmen, aiming at professionalization, has become a house with many interconnected rooms, where it is good living and working and were respectful difference of opinion, based on differences in practice and financial statute, successfully combines different visions under one large umbrella of professionalism. To maintain this unity is a challenge and a privilege for every three-year crew that guides this ship towards an even stronger future.

